# Effect of surgical stabilization of rib fractures in polytrauma: an analysis of the TraumaRegister DGU^®^

**DOI:** 10.1007/s00068-021-01864-0

**Published:** 2022-02-03

**Authors:** Lars Becker, Stefan Schulz-Drost, Christopher Spering, Axel Franke, Marcel Dudda, Rolf Lefering, Gerrit Matthes, Dan Bieler, Committee on Emergency Medicine, Intensive Care, Trauma Management (Sektion NIS) of the German Trauma Society (DGU)

**Affiliations:** 1 Department of Trauma Surgery, Hand and Reconstructive Surgery, University Hospital Essen, Essen, Germany; 2 Department of Trauma Surgery, Helios Hospital Schwerin, Schwerin, Germany; 3 Department of Trauma and Orthopedic Surgery, University Hospital Erlangen, Erlangen, Germany; 4 Department of Trauma Surgery, Orthopaedics and Plastic Surgery, University Hospital Göttingen Medical Center, Göttingen, Germany; 5 Department of Trauma Surgery and Orthopaedics, Reconstructive and Hand Surgery, Burn Medicine, German Armed Forces Central Hospital Koblenz, Koblenz, Germany; 6 Institute for Research in Operative Medicine (IFOM), Witten/Herdecke University, Cologne, Germany; 7 Department of Trauma Surgery and Reconstructive Surgery, Ernst Von Bergmann Hospital, Potsdam, Germany; 8 Department of Orthopaedics and Trauma Surgery, Heinrich Heine University Hospital, Düsseldorf, Düsseldorf, Germany

**Keywords:** Rib stabilization, Chest trauma, Rib fracture, Multiple trauma, SSRF

## Abstract

**Purpose:**

In severely injured patients with multiple rib fractures the beneficial effect of surgical stabilization is still unknown. The existing literature shows divergent results and especially the indication and the right timing of an operation are subject of a broad discussion. The aim of this study was to determine the influence of a surgical stabilization of rib fractures (SSRF) on the outcome in a multi-center database with special regard to the duration of ventilation, intensive care and overall hospital stay.

**Methods:**

Data from the TraumaRegister DGU^®^ collected between 2008 and 2017 were used to evaluate patients over 16 years with severe rib fractures (AIS ≥ 3). In addition to the basic comparison a matched pair analysis of 395 pairs was carried out in order to find differences and to increase comparability.

**Results:**

In total 483 patients received an operative treatment and 29,447 were treated conservatively. SSRF was associated with a significantly lower mortality rate (7.6% vs. 3.3%, *p* = 0.008) but a longer ventilation time and longer stay as well as in the intensive care unit (ICU) as the overall hospital stay. Both matched pair groups showed a good or very good neurological outcome according to the Glasgow Outcome Scale (GOS) in 4 of 5 cases. Contrary to the existing recommendations most of the patients were not operated within 48 h.

**Conclusions:**

In our data set, obviously most of the patients were not treated according to the recent literature and showed a delay in the time for operative care of well over 48 h. This may lead to an increased rate of complications and a longer stay at the ICU and the hospital in general. Despite of these findings patients with operative treatment show a significant lower mortality rate.

## Introduction

The surgical stabilization of rib fractures (SSRF) in severely injured patients is subject of increasing scientific discussion. Current publications by Swart et al. and Pieracci et al. suggested beneficial effects of a surgical stabilization of the fractured chest wall for the course of treatment [1, 2]. Other authors only stated an advantage of the surgical stabilization in the context of a flail chest or expressed themselves more cautiously with regard to the positive influence [3, 4]. A Cochrane analysis by Cataneo et al. showed some advantages in operated patients compared to the conservatively treated patient population, but pointed out the lack of sufficient sample sizes [5].

In a systematic review by De Jong et al. it was shown that supposedly more patients can benefit from SSRF than are currently treated surgically [6]. The data generally seem to show that in addition to the precise indication, the early timing of the operation is decisive whether the patient benefits. In the multicenter study by Pieracci et al., a daily increase of pneumonia and long-term ventilation were shown for patients with a flail chest who received no or a delayed surgical treatment[7]. The critical consideration of the treatment of serial rib fractures by Beks et al. on the other hand, stated in a retrospective multi-center evaluation that a general surgical treatment for patients with ≥ 3 rib fractures has no advantage [14]. Schulz-Drost et al. showed, in their TraumaRegister DGU^®^ work on the epidemiology of bony thoracic trauma in polytraumatized patients that the rate of operative reconstruction of the bony thorax increased with the severity of the injury. In particular, most patients with an operative treatment had an Abbreviated Injury Scale (AIS) of 4 or 5 [8].

In the light of the heterogeneous study situation with different indications and times of SSRF as well as small case numbers in most of the original research, the aim of this analysis is to evaluate the current status of the care of chest wall injuries in the TraumaRegister DGU^®^ and to show the associated differences between surgically and conservatively treated patients. The mortality rate, length of hospital stay, ICU-treatment and duration of intubation were defined as the primary endpoints. Parameters of the intensive care treatment and the survivors' outcome were defined as secondary endpoints.

## Materials and methods

The TraumaRegister DGU^®^ of the German Trauma Society (Deutsche Gesellschaft für Unfallchirurgie, DGU) was founded in 1993. The aim of this multi-center database is a pseudonymized and standardized documentation of severely injured patients.

Data are collected prospectively in four consecutive time phases from the site of the accident until discharge from hospital: (A) pre-hospital phase, (B) emergency room and initial surgery, (C) intensive care unit and (D) discharge. The documentation includes detailed information on demographics, injury pattern, comorbidities, pre- and inhospital management, course on intensive care unit, relevant laboratory findings including data on transfusion and outcome of each individual. The inclusion criterion is admission to hospital via an emergency room with subsequent ICU/ICM care or arrival at the hospital with vital signs and death before admission to an ICU.

The infrastructure for documentation, data management, and data analysis is provided by AUC—Academy for Trauma Surgery (AUC—Akademie der Unfallchirurgie GmbH), a company affiliated to the German Trauma Society. The scientific leadership is provided by the Committee on Emergency Medicine, Intensive Care and Trauma Management (Sektion NIS) of the German Trauma Society. The participating hospitals submit their data pseudonymized into a central database via a web-based application. Scientific data analysis is approved according to a peer review procedure laid down in the publication guideline of the TraumaRegister DGU^®^.

The participating hospitals are primarily located in Germany (90%), but a rising number of hospitals of other countries contribute data as well (at the moment from Austria, Belgium, China, Finland, Luxembourg, Slovenia, Switzerland, The Netherlands, and the United Arab Emirates). Currently, approx. 33,000 cases from more than 650 hospitals are entered into the database per year.

Participation in the TraumaRegister DGU^®^ is voluntary. For hospitals associated with TraumaNetzwerk DGU^®^, however, the entry of at least a basic data set is obligatory for reasons of quality assurance.

Patients aged 16 and older with rib fractures (AIS ≥ 3) from Germany and other European countries who were treated between 2008 and 2017. Only patients recorded with the standard data set were included. Since the reduced basic data set does not contain any information on operative care, patients documented with this data set were excluded. Patients with a minor thoracic trauma (AIS 0–2; that is, 1–2 fractured ribs) were excluded. Children under 16 years of age were also excluded, as were patients who were transferred to another hospital early after the initial trauma (< 48 h). In addition to the basic comparison of the groups of conservative treatment vs. surgical therapy, a matched pair analysis was carried out in order to sharpen the statement of any differences and to increase comparability. In order to obtain groups that were as comparable as possible, the surgically stabilized patients were paired with a conservatively treated patient with regard to the following criteria:

- age group (16–59, 60–69, 70–79 and older than 79 years)
- injury severity (AIS) in 4 body regions (head, thorax, abdomen, extremities)
- severity of the rib fracture (AIS 3/4/5)
- ventilation in the intensive care unit (yes / no)
- country of treatment (D, A, CH, B, NL).

To take the different influence of the injury pattern into account, pairs were matched using the AIS for four relevant body regions and for rib injuries. Each head, abdominal and extremity injury was assigned a counterpart depending on its severity. The matching categories, with regard to the severity of the injury, were defined with AIS 0–2, 3, 4 and 5 respectively. The procedure for rib injuries was analogous, with only AIS codes 3, 4, 5 being used here, since minor rib fractures (AIS 0–2) were excluded.

### Statistics

Primary endpoints were mortality and length of hospital stay, ICU-treatment and duration of intubation. Secondary endpoints were multi organ failure, time of operative stabilization of rib fractures and outcome according to the Glasgow outcome scale. Furthermore, general data of the patient collective, trauma mechanism, stabilization of rib fractures over time and age group distribution are presented.

The statistical evaluation was carried out with SPSS (Version 23, IBM Inc., Armonk, NY, USA). The data of the matched patients were compared with the aid of test procedures for dependent data (McNemar, Wilcoxon). The level of significance was set at 5% (*p* < 0.05). Missing values were not replaced, but excluded on a case-by-case basis. This study follows the current publication guidelines of the TraumaRegister DGU® and is registered under the TraumaRegister DGU^®^ project ID 2017–030.

## Results

**Fig. 1:**
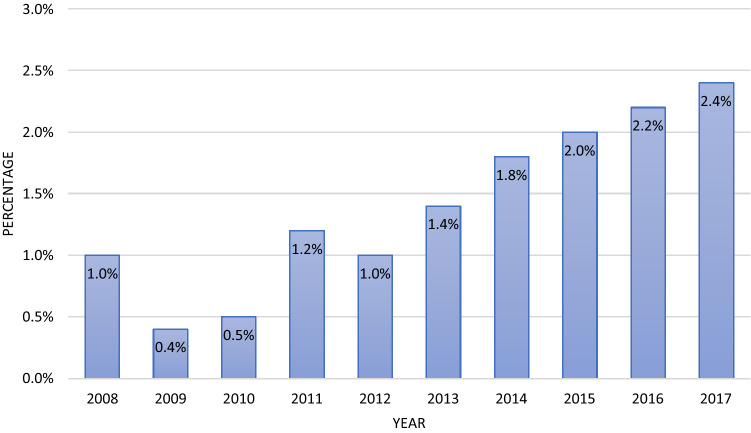
Stabilization of rib fractures over time in percent

**Fig. 2:**
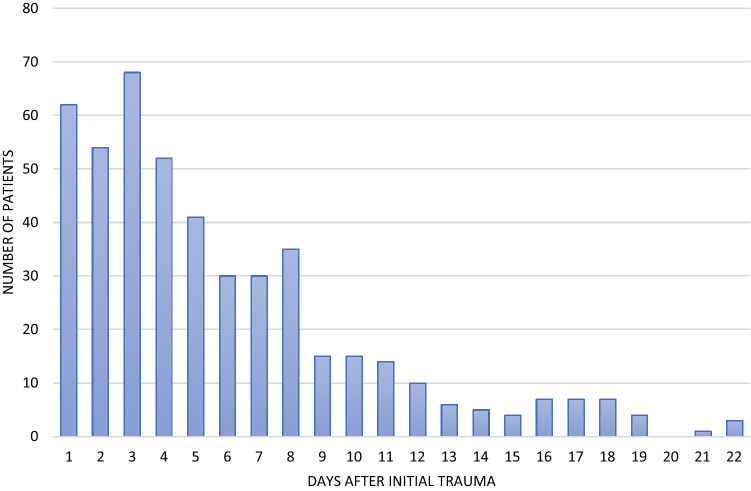
Time of operative stabilization of rib fractures in days after initial trauma (8 more patients had surgery > 22 days after trauma)

After application of inclusion and exclusion criteria *n* = 29.960 patients with a mean age of 55 years were included. 74% of the patients were male. The mean ISS was 26.8 and 86% were treated in a Level I trauma center. It should be noted that the number of minor thoracic trauma, as measured by AIS 3, predominates in the conservative group (63.3% vs. 23.8%). The total number of patients with surgical rib stabilization in the examined group was *n* = 483, those with conservative treatment of a chest wall injury was *n* = 29.447. (Table 1) During the observation period, SSRF increased in absolute and percentage terms. Ultimately, however, it remains a rarely performed procedure with less than 2% of all chest wall injuries with at least 3 broken ribs (Fig. 1). The timing of care is concentrated in the first few days after the trauma. Over three quarters of all operations are performed within the first eight days after trauma (Fig. 2).

**Table 1:** Patient collective—conservative treatment vs. surgical stabilization

	Patient collective, total	
	Conservative treatment (n = 29,477)	Surgical Stabilization of rib fractures (n = 483)
General data		
Age (years)	54.9 (SD 18.3)	58.2 (SD 15.4)
Male (n)	21846 (74.3%)	378 (78.3%)
ISS	26.8 (SD 13.4)	27 (SD 11.2)
Level I (*n*)	25202 (85.5%)	426 (88.2%)
Level II (*n*)	3486 (11.8%)	50 (10.3%)
Level III (*n*)	789 (2.7%)	7 (1.4%)
Primarily treated patients (*n*)	26025 (88.3%)	400 (82.8%)
transferred patients (*n*)	3452 (11.7%)	83 (17.2%)
Chest trauma		
AIS 3	18757 (63.3%)	115 (23.8%)
AIS 4	6151 (20.9%)	235 (48.7%)
AIS 5/6	4569 (15.5%)	133 (27.5%)
Treatment		
Duration of intubation (d)	5.1 (SD 9.9) Median 1	9.5 (SD 12.3) Median 4
Duration of ICU treatment (d)	9.5 (SD 12.3) Median 4	16.1 (SD 15.7) Median 11
Hospital stay (d)	21.0 (SD 20.4) Median 16	29.3 (SD 18.7) Median 25
Outcome		
Organ failure (single; *n*)	11112 (43.3%)	273 (61.6%)
Multi organ failure (*n*)	7267 (28.2%)	185 (41.7%)
Died, total (*n*)	4147 (14.1%)	22 (4.6%)
Died within 24 h (*n*)	2024 (6.9%)	0
Died within 48 h (*n*)	2305 (7.8%)	0

*ISS* Injury Severity Score, *AIS* Abbreviated Injury Scale, *ICU* Intensive Care Unit

Table 1 gives an overview of the patient groups examined. Both groups showed similar serious injuries as measured by the Injury Severity Score (ISS), the operative group showed a significantly lower mortality rate. (conservative 7.6% vs. 3.3%, *p* = 0.008). In contrast, the length of stay in the intensive care unit (9.5 days vs. 16.1 days) and the overall hospital stay are (21 days vs. 29.3 days) longer in the operative group (*p* > 0.001). Furthermore, the duration of intubation was longer and more patients had single organ failure or multiple organ failure to the detriment of the operative group in the overall group. In the surgically treated group, over 76.2% of the patients had a severe chest trauma (AIS 4 & 5).

The matched-pair analysis was carried out to ensure the best possible comparability of many patients. 395 patients with SSRF could be matched with a respective patient without such intervention. The respective matched pairs subgroups showed comparable values for age, sex, ISS, trauma center level and injury mechanism. Lung lacerations are more than twice as frequent in the surgical group (conservative 4.8% vs. surgical 10.9%; *p* = 0.001). However, there was no significant difference in the presence of lung contusion (42.3% vs. 46.1%). As for the collective as a whole, relevant differences could be detected for the duration of intensive care and hospital treatment as well as for singular and multiple organ failure. A significantly longer duration in the surgical group was found for all 3 parameters (Table 2).

**Table 2:** Matched pairs groups: conservative treatment vs. surgical stabilization

Matched-Pairs-cohort (*n* = 395 pairs)			
	Conservative treatment	Surgical stabilization of rib fractures	
Age (years)	54.8 (SD 16.9)	56.8 (SD 15.1)	*p* = 0.10
Male (*n*)	312 (80%)	316 (80%)	*p* = 1.00
ISS	25.6 (SD 10.3)	25.4 (SD 10.3)	*p* = 0.69
RISC-prognosis (only primarily treated)	11.8%	10.2%	*p* = 0.27
Level I	346 (87.6%)	338 (85.6%)	*p* = 0.59
Level II	41 (10.2%)	50 (12.7%)	
Level III	8 (2.0%)	7 (1.8%)	
Trauma mechanism			
Blunt	377 (99.5%)	368 (98.7%)	*p* = 0.25
Penetrating	2 (0.5%)	5 (1.3%)	
Age group distribution			
16–59 years	229 (58%)		
60–69 years	76 (19.2%)		
70–79 years	68 (17.2%)		
≥ 80 years	22 (5.6%)		
Injuries			
AIS head ≥ 3	73 (18.5%)		
AIS abdomen ≥ 3	36 (9.1%)		
AIS extremities ≥ 3	78 (19.7%)		
AIS thorax = 3	110 (27.8%)		
AIS thorax = 4	184 (46.6%)		
AIS thorax = 5	101 (25.6%)		
AIS ribs = 3	159 (40.3%)		
AIS ribs = 4	156 (39.5%)		
AIS ribs = 5	80 (20.3%)		
Thoracic injury only	236 (59.7%)	238 (60.3%)	*p* = 0.89
Lung contusion	167 (42.3%)	182 (46.1%)	*p* = 0.28
Lung laceration	19 (4.8%)	43 (10.9%)	*p* = 0.001
Treatment			
Duration of intubation (d)	6.9 (SD 9.3) M 2	9.6 (SD 12.1) M 4	*p* = 029
Duration of ICU treatment (d)	11.9 (SD 11.8) M 8	16.2 (SD 15.4) M 12	*p* < 0.001
Hospital stay (d)	25.3 (SD 21.1) M 20	29.3 (SD 17.9) M 25	*p* < 0.001
Outcome			
Organ failure (single; *n*)	185 (51.0%)	219 (60.7%)	*p* = 0.009
Multi organ failure (*n*)	103 (28.0%)	144 (39.8%)	*p* = 0.001
Died	30 (7.6%)	13 (3.3%)	*p* = 0.008
Discharged home	184 (46.8%)	192 (48.6%)	*p* = 0.084
Rehab-clinic	114 (29.0%)	124 (31.4%)	
Transfer to another hospital	51 (13.0%)	56 (14.2%)	
Other	14 (3.6%)	10 (2.5%)	
Glasgow Outcome Scale (survivors)			
Persistent vegetative status	6 (1.7%)	5 (1.3%)	*p* = 0.086
Severe disability	34 (9.6%)	59 (15.9%)	
Moderate disability	96 (27.2%)	100 (26.9%)	
Good recovery	217 (61.5%)	208 (55.9%)	

*ISS* Injury Severity Score, *RISC* Revised Injury Severity Classification, *AIS* Abbreviated Injury Scale, *ICU* Intensive Care Unit

**Fig. 3:**
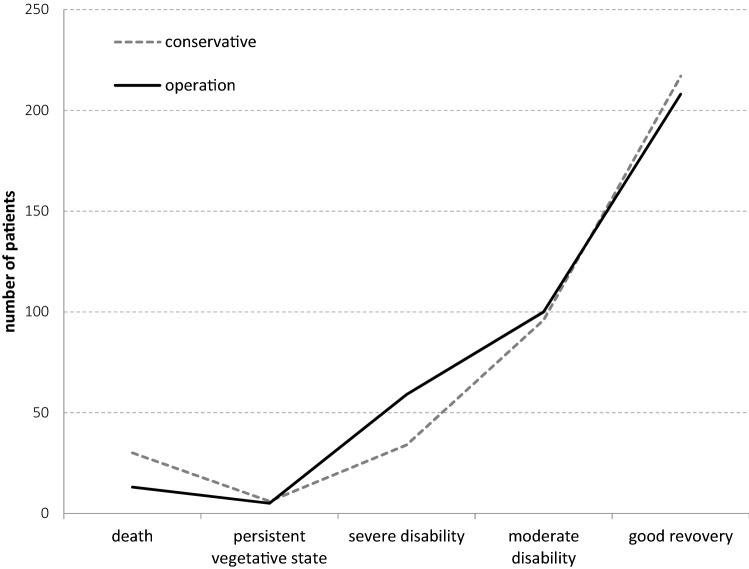
Outcome of the matched pairs groups using the Glasgow Outcome Scale

Looking at the result of both matched pair groups using the Glasgow Outcome Scale (GOS, Fig. 3) it could be shown that in both groups in 4 of 5 cases a good or very good outcome (conservative 88,7%, SSRF 82,8%) could be achieved. In contrast, patients who were assessed as severely disabled at discharge are more common (conservative 9.6% vs. 15.9). When comparing the two groups, there are no differences in terms of follow-up care after hospital treatment.

## Discussion

Stable, nondisplaced rib fractures can usually be treated conservatively without any problems. There is also broad consensus on this in the literature. For the treatment of unstable chest wall injuries ("flail chest"), however, optimal care and the advantages or disadvantages of surgical treatment have been discussed for a long time. In addition to an international consensus statement (Pieracci et al.), there are no national or international guidelines available so far and a comparison of the literature is elusive by very inconsistent treatment strategies [9–14].

In principle, the surgical stabilization of displaced rib fractures is a suitable means and a method that has been known for decades to achieve a reconstruction of the chest wall and the restoration of adequate respiratory mechanics while reducing pain at the same time [15]. Recent studies have shown a positive effect on survival and outcome [16]. Nevertheless, it is still unclear whether a demonstrably positive effect can be achieved at with a surgical stabilization and for which patients overall or for which parameters there is a benefit [17].

The significant lower mortality that can be demonstrated in the present study for patients undergoing surgical stabilization, both in the total collective (4.6% vs. 14.1%) and in the matched pair subgroup (3.3% vs. 7.6%), corresponds to a large number of studies carried out in recent years. E.g. DeFreest et al. showed in their study, also carried out as a matched pair analysis, a lower mortality of 2.4% vs. 11.1% and demonstrated a similar positive effect from surgical treatment [15]. The meta-analysis´ by Beks et al. and Liu et al. were able to show a significantly lower mortality rate for the group of operated patients. The determined risk ratio of mortality in Beks publication was 0.41, the odds ratio for mortality stated by Liu was 0.28. Both included several randomized and controlled studies [18, 19].

In contrast, review articles such as the Cochrane analysis by Cataneo et al. as well as the systematic review of existing review articles by Ingoe et al. could not prove any survival advantage for surgical stabilization of unstable chest injuries. The predominantly low level of evidence of the available studies was criticized as a limiting factor in both papers [5, 17].

Almost the entire existing literature regarding SSRF is based on a patient population from controlled studies. This, due to its artificial framework and patient selection, may lead to a bias of the beneficial effects of an operative stabilization. In our matched pairs analysis we were able to confirm this positive effect for patients with a severe thoracic trauma in a large, multicentered and unselected population based cohort for the first time.

In addition to the lower mortality rate, the Glasgow Outcome Scale showed a slightly better, non-significant, outcome for patients after surgical treatment. The rate of slightly disabled and well-recovered patients was unchanged in comparison. De Moya et al. came to similar results without evidence of a relevant improvement in outcome as well as the study by Cataneo and Marasco. Pieracci et al. on the other hand showed a daily increasing risk of approx. 30% for pneumonia, 27% for long-term ventilation and 26% for tracheotomy with unstable thorax without surgery. In accordance with this, the tendency towards the advantage of the operative group is described predominantly in the first weeks after trauma, but so far there is no reliable evidence of a long-term improvement in outcome compared to non-operative treatment in the literature [5, 20–22]. Most of the patients in this study were operated significantly later than the recommended 48 h after trauma. This may have masked a potential benefit of surgical care.

Our analysis showed a significantly longer duration of ventilation time, the length of stay in the intensive care unit, and the total hospital stay, than in most publications.

These prolonged times could be seen in the data set of the TraumaRegister DGU^®^ both in the overall collective and in the matched pair analysis for the operative treatment. In the data analysis, however, no explanation could be found in the data set for this observation. These results are in contrast to almost all available studies, which were able to demonstrate a significant reduction in the respective times for all three parameters [1, 14, 17, 18, 23–29]. However, some studies were also able to show similar results with longer ventilation and length of stay [15, 20, 21]. Contrary to the current recommendations in the literature, a delay in the time for operative care of well over 48 h in the examined collective could represent a possible cause in combination with the then known increased complications (pneumonia rate, long-term ventilation, increased tracheostomy rate). This will be the subject of further investigations by our working group.

There is a possible bias in the data set of the TraumaRegister DGU^®^ that many hospitals do not (yet) carry out surgical stabilization in the examined period 2008–2017 according to the recommended indications and time of surgery from the literature of the last years, but rather in patients with a difficult course and prolonged weaning. These patients more likely show a rather poor outcome overall and therefore no difference can be demonstrated.

In addition, it cannot be tracked whether and, if so, at what point after the initial trauma the indication for a stabilization of the chest wall was considered. In addition, patients who died early or who were moribund were mostly not operated on and are therefore assigned to the non-operative group.

These factors may contribute to the longer time of intubation and intensive care treatment as well as the longer hospital stay in the operated group.

While the literature recommends surgery after 24–48 h, we see a significantly later point in time in the present collective. It is therefore to be expected that the operation will result in a “second hit” for the patient who will subsequently have to remain in the intensive care unit for a longer period of time before he finally recovers.

### Limitations of the study

The data set used by the Trauma Register DGU^®^ leads to several methodological limitations from the outset. First of all the analyzed data set is retrospective and was not specifically designed to obtain the extent of thoracic injuries. The localization and morphology of the rib fractures and lung injuries are so far not covered by the AIS classification. The morphological classification of the fractures, however, plays an important role in determining the indication for a surgical treatment. Complicating matters only the duration of a mechanical ventilation is documented, no information about different parameters regarding the ventilation can be obtained from the data set. Nor can it be subsequently clarified whether the indication for SSRF was based on radiological diagnostics or on functional parameters. The assessment of the outcome by the Glasgow Outcome Scale is also only roughly indicative and does not make any statements about relevant thoracic outcome parameters such as restriction, pain, deformity or nonunions. In general the choice of diagnostic means leading to the classification of thoracic injuries is not clearly defined and the respective interpretation is strongly dependant on the individual examiner and the quality of the submitted data in the registry. Further studies with a data pool specifically designed for thoracic trauma would be necessary to clarify these questions.
